# What are the similarities and differences in structure and function among the three main models of community health centers in China: a systematic review

**DOI:** 10.1186/s12913-015-1162-z

**Published:** 2015-11-10

**Authors:** Haitao Li, Dongfu Qian, Sian Griffiths, Roger Yat-nork Chung, Xiaolin Wei

**Affiliations:** School of Medicine, Shenzhen University, Shenzhen, China; School of Health Policy and Management, Nanjing Medical University, Nanjing, China; School of Public Health and Primary Care, Faculty of Medicine, The Chinese University of Hong Kong, Hong Kong, China; Dalla Lana School of Public Health, University of Toronto, Toronto, Canada

**Keywords:** Primary care, Community health service, Community health center, Model, Systematic review

## Abstract

**Background:**

There are three major models of primary care providers (Community Health Centers, CHCs) in China, i.e., government managed, hospital managed and privately owned CHCs. We performed a systematic review of structures and health care delivery patterns of the three models of CHCs.

**Methods:**

Studies from relevant English and Chinese databases for the period of 1997–2011 were searched. Two independent researchers extracted data from the eligible studies using a standardized abstraction form. Methodological quality of included articles was assessed with the Mixed Methods Appraisal Tool (MMAT).

**Results:**

A total of 13 studies was included in the final analysis. Compared with the other two models, private CHCs had a smaller health workforce and lower share of government funding in their total revenues. Private CHCs also had fewer training opportunities, were less recognized by health insurance schemes and tended to provide primary care services of poor quality. Hospital managed CHCs attracted patients through their higher quality of clinical care, while private CHCs attracted users through convenience and medical equipment.

**Conclusions:**

Our study suggested that government and hospital managed CHCs were more competent and provided better primary care than privately owned CHCs. Further studies are warranted to comprehensively compare performances among different models of CHCs.

## Background

The greatest discontents reflected by the public in China are the difficult access to health care, and impoverishment due to heavy medical expenses, which are commonly known respectively in Chinese as “*Kan bing nan, and “Kan bing gui*” [1]. It has been shown that over one third of households have reduced their consumption or been impoverished by health-related expenditures in China [1]. In addition, significant inequalities exist in health care utilization and health care outcomes across regions, between rural and urban areas, and across populations with different socioeconomic statuses [2, 3]. Primary care is widely considered to be the cornerstone of a health system, since it brings improved access to essential health services, puts heavier focus on prevention and initiates early management of health problems, which lead to better health outcomes and less health inequity in the population [4–9]. In 2009, China launched its comprehensive health reform plan aiming to build a more equitable and efficient health system through a stronger primary care system [10].

Primary care in China is mainly provided by Community Health Centers (CHCs) in urban areas and township hospitals in rural areas. Due to the rapid urbanization, most township hospitals were also called CHCs in order to respond the rapidly rising health needs in emerging towns. CHCs were firstly established by government or public hospitals in 1997. In 1999, government began to promote privately owned CHCs due to the inadequate public funding. Since then, there has been a rapid expansion in the number of CHCs in China. The proportion of cities with CHCs offering primary care to the public reached over 90 % in 2009 with a total number of 27,308 CHCs [11]. There are three major models of CHCs in terms of ownership and operation, i.e., government managed, hospital managed, and privately owned CHCs [12]. Government managed and hospital managed CHCs accounted for the majority of all CHCs (77 % in 2007) [13], while privately owned CHCs accounted for 9 % and were mainly located in the western and southern parts of China [13].

Government managed CHCs are independent entities fully funded and directly supervised by the local governments. The governments thus assume greater responsibilities in supporting and promoting CHCs’ development. In addition, these CHCs are independent of public hospitals. The majority of CHCs with this model were converted either from former first-level hospitals or from the out-reach clinics of secondary and tertiary hospitals. Their functions were transformed from providing clinically focused services to providing combined and integrated primary care services, which included clinical care, preventive medical care, health education and promotion, rehabilitation, technical support for family planning, as well as chronic disease management.

Hospital managed CHCs are established directly by a hosting public hospital. Since public hospitals are owned by the government, CHCs of this model are indirectly owned by governments, but directly operated and managed by the hosting hospitals as non-independent entities, often as a department or an out-reach clinic. Hosting hospitals play an essential role in financing, managing and supervising of these CHCs. For example, government funding is allocated to the CHCs via their hosting hospitals.

Privately owned CHCs are private businesses who take full responsibility for profits and losses. They receive partial financial support from the government for the provision of public health services for the relatively permanent populations in the catchment area. Similar to government managed ones, private CHCs are also independent of public hospitals.

The different ownership types of CHCs determine to whom and for what they are held accountable, which would consequently influence the delivery of community health services (i.e., the delivery of primary care services), and in turn, the health status and satisfaction of the populations being served [14]. In recent years, there has been a lively debate regarding the preferred primary care model as a regular source of care for solving the problem of inefficient use of medical resources. However, there has been limited evidence in China or elsewhere concerning the impact of these ownership types on delivery by different primary care providers. We therefore performed this systematic review to identify the differences among the three models of CHCs in terms of their structure and primary care service delivery, to show relative attributes and benefits of different models of CHCs, which was expected to provide valuable feedback to policy makers for establishing a better primary care-led healthcare system in China and similar settings.

## Methods

We conducted a systematic review according to the following strategies. The Preferred Items for Systematic Reviews and Meta-Analyses (PRISMA) guidelines were followed to report the review process.

### Search criteria

We retrieved literature from the English databases of MEDLINE and EMBASE, and the Chinese databases of CNKI, VIP and WANFANG, which were published from January 1997 to September 2011. We chose the year 1997 as the starting year because it was the time when the community health service system was established in China. The searching terms included “Community Health Service”, “Community Health Center”, “Community Health Station”, “Community Health Organization”, “primary care” or “primary health care” and a search for “ownership”, “source”, “model”, “type”, “government”, “hospital” or “private”. The specific search strategy was shown in Table 1. References listed in published articles such as literature reviews were screened to identify additional sources not identified in the database searches.

**Table 1:** Search terms and search strategy

1 Community health services (MeSH) 2 Community health service* 3 Community Health Centers (Mesh) 4 Community Health Center* 5 Community Health Station 6 Community Health Organization 7 primary health care (MeSH) 8 primary care 9 ownership (MeSH) 10 source* 11 model* 12 type* 13 government* 14 hospital* 15 private* (1 or … to 8) AND (9 or … to 15)	

*Treated as a placeholder for any unknown term(s)

### Study selection criteria

#### Language and location

Only papers published in Chinese or English were included in the study. We confined the country to China only, but the study location was not restricted, meaning that the study could be at a national or district level.

#### Study designs

We included both cross-sectional and longitudinal studies. Literature reviews in research papers were also included. Articles such as opinion pieces, letters, news, commentaries, editorial, meeting abstracts and bibliographies were excluded as no research evidence was provided.

#### Target of studies

We looked for literature that investigated differences among the three models of CHCs, i.e., government managed, hospital managed or privately owned CHCs. The comparison could be among all of the three models, between any two of them, between a combination of any two with the other, or among primary, secondary and tertiary hospital managed CHCs.

### Selection of publications

Two reviewers independently evaluated the relevance of each article by considering the title and abstract. Subsequently, the full texts of the papers identified as potentially relevant were obtained. If we were uncertain whether or not the paper qualified for inclusion, we nonetheless included it and obtained the full text. Uncertainties were then resolved by discussion between the two reviewers. According to the eligibility criteria, we then screened all the full texts to select the relevant studies.

### Data extraction

A standardized data extraction form was formulated by the research team. Two researchers independently performed data extraction. One researcher was responsible for the initial data extraction, while the other was in charge of verifying the data extracted by the first researcher. The information extracted from the selected studies included main authors, study design, time period of data, data sources and contents of the study. To frame the analysis, any of the following issues of CHCs that were addressed by the included papers were extracted: 1) structure, which referred to a collection of organizational factors of CHCs that defined how the primary care services were provided, including human resources, financing aspects and premises; 2) delivery of primary care services including community health service utilization and quality; and 3) outcome measured by public satisfaction.

### Quality assessment

All included articles were independently assessed for methodological quality by the two reviewers. We used adapted Mixed Methods Appraisal Tool (MMAT) for quality assessment [15]. This tool was judged suitable to be used in mixed method reviews, being with substantial validity and reliability. The MMAT used in our study contained three sets of criteria: 1) a “qualitative” set for qualitative studies, and qualitative components of mixed methods research; 2) an “observational descriptive” set for observational descriptive quantitative studies, and observational descriptive components of mixed methods research; and 3) a “mixed methods” set for mixed methods research studies. Each study type was judged within its methodological domain. For each criterion, the presence/absence was scored 1 and 0, respectively [16]. Discrepancies in quality assessment between reviewers related primarily to how findings related to researchers’ influence and sampling strategy of quantitative data, and were settled through consensus. A quality score for each article was then calculated through dividing the total points scored by the total points possible. Each article was classified as weak (≤0.50), moderate-weak (0.51 to 0.65), moderate-strong (0.66 to 0.79), or strong (≥0.80) in terms of study quality [17].

## Results

**Fig. 1:**
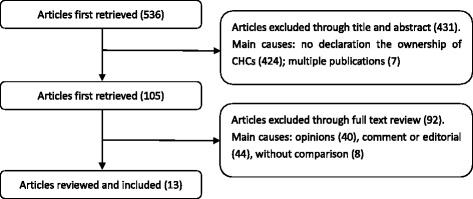
Shows the study selection process. Of the initially yielded 536 papers, we firstly excluded 431 ones by reviewing titles and abstracts, arriving at 105 articles with full texts. Among the 105 articles, 13 met the inclusion criteria and formed the basis of the findings. All the 13 articles were of cross-sectional study design and were in Chinese

The initial search yielded 536 papers. Following the selection process as shown in Fig. 1, we first excluded 431 articles after reviewing titles and abstracts, which arrived at 105 articles with full texts. Among these 105 articles, 13 met the inclusion criteria and formed the basis of the findings. All the 13 articles were of cross-sectional study design and were in Chinese, among which 6 were conducted at the national level and the others were at either the provincial or municipal level. Of all the 13 included papers, five studies compared among the three models of CHCs, five compared combined government managed and hospital managed CHCs with privately owned CHCs, and three compared among primary, secondary and tertiary hospital managed CHCs (Table 2).

**Table 2:** Characteristics of the included studies

Main author	Comparison	Study area	Study design	Data sources	Contents of the study		
					Structure	Delivery of health services	Public satisfaction
Zhao et al. (2010) [26]	G vs. H vs. P	National	Cross sectional	Survey and interview	√	√	√
Wang (2008) [13]	G + H vs. P	National	Cross sectional	Survey and interview	√	√	
Guo (2010) [21]	G vs. H vs. P	Guangdong province	Cross sectional	Survey and interview	√	√	√
Zhang (2008) [23]	G vs. H vs. P	Jiangsu province	Cross sectional	Survey and interview	√		
Li (2009) [30]	H1 vs. H2	Shenzhen	Cross sectional	Survey and interview	√		
Fan et al. (2007) [18]	G + H vs. P	Heilongjiang	Cross sectional	Survey	√		
Yao et al. (2010) [27]	G vs. H vs. P	National	Cross sectional	Survey and interview			√
Li et al. (2010) [25]	G + H vs. P	National	Cross sectional	Survey	√		√
Yang & Dai (2010) [22]	G + H vs. P	Chengdu City	Cross sectional	Survey	√	√	
Chen & Du (2010) [24]	H1vs. H2 vs. H3	Beijing City	Cross sectional	Survey	√	√	
Wang et al. (2009) [28]	G + H vs. P	National	Cross sectional	Survey			√
Xu et al. (2011) [19]	G vs. H vs. P	National	Cross sectional	Survey	√	√	
Chen et al. (2009) [29]	H1vs. H2 vs. H3	Beijing City	Cross sectional	Survey			√

*Note*: *G* government managed CHCs, *H* hospital managed CHCs, *P* privately owned CHCs, *H1* primary hospital managed CHCs, *H2* secondary hospital managed CHCs, *H3* tertiary hospital managed CHCs

Table 3 shows the methodological quality of the included articles. Of the included reports, 5 were rated as strong, 7 as moderate-strong, and 1 as moderate-weak, respectively. The validity and reliability of research measures used were not reported or were “assumed” to be valid and reliable based on their own subjective judgment in the majority of these articles. Nonetheless, they were widely recognized as effective measures (Table 3).

**Table 3:** Quality assessment of the included studies using Mixed Methods Appraisal Tool (MMAT)

Author(s), Year	Qualitative				Quantitative descriptive				Mixed methods			Total Points	Score	Quality
	Sources of data relevant to objectives	Analysis process relevant to objectives	Consideration of findings relate to context	Consideration of findings relate to researchers’ influence	Sampling strategy relevant to objectives	Sample representativeness	Measurements appropriate	Acceptable response rate	Mixed methods research design relevant to objectives	Integration of results relevant to objectives	Consideration of limitations associated with this integration			
Zhao et al. (2010) [26]	1	1	1	0	1	1	1	1	1	0	0	8/11	0.73	Moderate-Strong
Wang (2008) [13]	1	1	1	0	1	1	1	1	1	0	0	8/11	0.73	Moderate-Strong
Guo (2010) [21]	1	1	1	0	1	1	1	1	1	0	0	8/11	0.73	Moderate-Strong
Zhang (2008) [23]	1	1	1	0	1	0	1	1	1	0	0	7/11	0.64	Moderate-Weak
Li (2009) [30]	1	1	1	0	1	1	1	1	1	0	0	8/11	0.73	Moderate-Strong
Fan et al. (2007) [18]	N/A	N/A	N/A	N/A	1	1	1	1	N/A	N/A	N/A	4/4	1.00	Strong
Yao et al. (2010) [27]	1	1	1	0	1	1	1	1	1	0	0	8/11	0.73	Moderate-Strong
Li et al. (2010) [25]	N/A	N/A	N/A	N/A	1	1	1	1	N/A	N/A	N/A	4/4	1.00	Strong
Yang & Dai (2010) [22]	N/A	N/A	N/A	N/A	1	1	1	1	N/A	N/A	N/A	4/4	1.00	Strong
Chen & Du (2010) [24]	N/A	N/A	N/A	N/A	1	0	1	1	N/A	N/A	N/A	3/4	0.75	Moderate-Strong
Wang et al. (2009) [28]	N/A	N/A	N/A	N/A	1	1	1	1	N/A	N/A	N/A	4/4	1.00	Strong
Xu et al. (2011) [19]	N/A	N/A	N/A	N/A	1	1	1	1	N/A	N/A	N/A	4/4	1.00	Strong
Chen et al. (2009) [29]	N/A	N/A	N/A	N/A	1	0	1	1	N/A	N/A	N/A	3/4	0.75	Moderate-Strong

### Structure

We identified differences in operation structure on the aspects of financing, premises and human resources.

#### Financing

The revenues of CHCs in China came from three major sources: government funding, health insurance reimbursement, and out-of-pocket payments from patients. Overall, three studies focused on the financing of the different models of CHCs [13, 18, 19].

Two studies provided information regarding the proportion of government funding in total CHC revenue [13, 18]. Both studies showed that government funding accounted for a higher proportion in government managed and hospital managed CHCs than privately owned CHCs. One study in 29 cities across China pointed out that in 2007 government funding accounted for less than 7 % of the total revenue of privately owned CHCs, while the proportion was 19 % for that of the other two models [13]. Another study conducted in Harbin city of the Heilongjiang Province showed that government funding accounted for 25 % of total revenues in government and hospital managed CHCs, but privately owned CHCs did not receive any government funding at all [18].

Two studies described whether services in CHCs had been covered by health insurance schemes [13, 19]. Along with the rapid expansion of health insurance schemes including the Basic Medical Insurance for Urban Employees and the Basic Medical Insurance for Urban Residents, more CHCs had been covered by health insurance schemes over time [20]. The study in 29 cities of China showed that 34 % of privately owned CHCs and 43 % of government and hospital managed CHCs were covered by insurance schemes [13]. Another study found the coverage rate was 62 % for privately owned CHCs, 66 % for government managed CHCs and 79 % for hospital managed CHCs [19].

Nevertheless, government funding and health insurance reimbursement were insufficient to support running CHCs in all the three models. Patients’ out-of-pocket payments for medical services and drugs remained the major source of revenue for CHCs. The share of out-of-pocket payments accounted for 57 % in privately owned CHCs, and 51 % in the other two models of CHCs [13].

#### Premises

Four studies described premises in different models of CHCs [13, 18, 21, 22]. Two studies found that privately owned CHCs tended to rent their offices while the other models of CHCs tended to own their own offices [13, 21]. A study in Guangdong province showed that nearly 97 % of the offices of privately owned CHCs were rented in 2009, while the percentages were 57 and 22 % for hospital managed and government managed CHCs, respectively [21]. Wang’s study found that 77 % of the offices in the private CHCs were rented in 2007 which was higher than that of the other two models of CHCs [13].

Regarding the building area of CHC offices, we did not draw a conclusion from the four included studies. Of the four studies, two showed that the building area of privately owned CHCs was larger than that of the other two models of CHCs [13, 18], while the other two studies showed opposite results [21, 22].

#### Human resources

Overall, seven studies addressed the human resources of the different models of CHCs [13, 18, 21–25]. The CHC workforce has expanded rapidly during the last decade. The number of doctors increased from 17,281 in 2003 to 109,734 in 2009, and the number of nurses grew from 12,484 to 79,711 during the same period [12]. Privately owned CHCs employed fewer health workers compared to the other two models of CHCs [13, 22]. Moreover, fewer training opportunities of primary care were provided to doctors and nurses working at privately owned CHCs than those working at the other two models of CHCs [13].

The education level of the entire health workforce among the three models of CHCs remained low compared to that in hospitals. Most of doctors and nurses in CHCs had only received 3-year medical training from medical colleges, or equivalent training from secondary schools, while most doctors in hospitals had a 5 year bachelor degree from medical universities. We did not draw any conclusion from the six studies regarding the differences in the education level of health workers among the different models of CHCs (Table 4). Three studies indicated that the general education level of health workers in privately owned CHCs was lower than that in the other two models of CHCs [21–23]. The study in Guangdong province showed that about 61 % of the health workforce graduated from technical secondary school, as compared to about one third in the other two models of CHCs [21]. Most health workers in privately owned CHCs in Chengdu had only reached a secondary school education level, while about one third in government managed and hospital managed CHCs had college education levels [22]. The proportion of health workers with an undergraduate education level in privately owned CHCs in Jiangsu province and Chengdu city was lower than the other two models of CHCs [23]. However, the study by Li and colleagues [25] at a national level and the study by Fan and colleagues [18] in Heilongjiang province found that the education level of health workers in privately owned CHCs was relatively higher than that in government managed and hospital managed CHCs. Among hospital managed CHCs, the education level of health workers in tertiary hospital managed CHCs was higher than that in primary and secondary managed CHCs [24].

**Table 4:** The percentage of health workers with different education level and professional title

First author	Comparison	Education level			Professional title		
		>5-yearundergraduate	3-year college	<technical secondary school	Senior	Middle	Junior
Guo Haixiu	G	16.1	48.0	30.3	0.2	8.6	62.2
	H	21.6	40.8	37.2	6.0	23.4	64.0
	P	11.4	25.2	61.2	3.1	16.7	53.7
Zhang Heping	G	20.0	36.7	40.0	10	23.3	26.7
	H	15.2	27.2	48.5	9.1	27.3	24.2
	P	7.4	29.7	48.1	0.0	3.7	22.2
Li Yongbin	G + H	27.96	38.33	33.71	11.49	36.66	51.85
	P	31.66	43.93	24.41	20.27	44.48	35.25
Fan Lihua	G + H	21.2	33.6	45.4	18.9	32.0	49.1
	P	9.6	69.8	20.6	32.9	50.7	16.4
Yang Dehua	G + H	20.7	33.6	30.2	10.0	22.5	58.0
	P	13.4	39.4	42.6	10.5	29.4	52.8
Chen Jie	H1	20.0	43.0	37.0	4.4	34.8	60.8
	H2	19.3	42.2	38.5	6.4	41.3	52.3
	H3	35.1	37.7	27.2	14.0	38.6	47.4

*Note*: *G* government managed CHCs, *H* hospital managed CHCs, *P* privately owned CHCs, *H1* primary hospital managed CHCs, *H2* secondary hospital managed CHCs, *H3* tertiary hospital managed CHCs

The health workforce in China is categorized into junior, middle and senior titles, which indicate their professional competency. The results regarding the differences in the professional titles of health workers among the three models of CHCs were inconsistent. Three studies in different places in China found that the professional titles of health workers in the privately owned CHCs were generally higher than in the government managed and hospital managed CHCs [18, 22, 25], while the study in Jiangsu province found that the professional titles of health workers in privately owned CHCs were the lowest among all three models of CHCs [23]. The study in Guangdong province found that the professional title of health workers in hospital managed CHCs was the highest among all three models of CHCs [21]. Furthermore, the professional title was found to be the highest for tertiary hospital managed CHCs among primary, secondary and tertiary hospital managed CHCs [24, 30].

### Primary care delivery

Overall, six studies had focused on the delivery of primary care services [13, 19, 21, 22, 24, 26].

#### Output of primary care

Patients were more likely to seek health care services from hospital managed CHCs than the other two models of CHCs. Hospital managed CHCs were found to have better output of clinical care in terms of the average number of outpatient services per physician (i.e., the number of outpatient services that a physician offers) compared with CHCs of the other two models in Guangdong province [21]. The national study by Xu and colleagues [19] showed similar results. Additionally, among the CHCs operated by hospitals with different levels, tertiary hospital managed CHCs were the most preferred by patients. Chen and Du [24] showed that 76 % of community residents in Beijing had ever used the primary care services of CHCs managed by tertiary hospitals, 61 % had used the services of CHCs managed by secondary hospitals, and 58 % had used the services of CHCs managed by primary hospitals.

Inconsistent results were found, when comparing the different models of CHCs, with respect to the hypertension management rates, i.e., the proportion of hypertensive patients under the standard hypertension management out of the total number of hypertensive patients identified in the community. Two studies, one in Guangdong province and the other at the national level, showed that the hypertension management rate was the highest for the privately owned CHCs, followed by hospital managed CHCs and then government managed CHCs [19, 21]. But another national study showed that the proportion was the lowest in privately owned CHCs [13]. In terms of health education, tertiary hospital managed CHCs were found to provide the best health education evaluated by patients compared to other hospital managed CHCs in Beijing [24]. Privately owned CHCs had the poorest vaccination rate in children when compared with government managed and hospital managed CHCs [19].

#### Quality of care

The quality of clinical and public health services provided by private CHCs tended to be poorer than that of the other two models of CHCs. For example, more irrational use of drugs defined by the number of drugs prescribed, percentage encounters with antibiotics prescribed, or percentage encounters with injections prescribed, had been observed among privately owned CHCs than the other models of CHCs [13]. Almost 95 % of all CHCs had begun to establish health records for community residents, but the quality of health records (i.e., the completeness and accuracy) established by the hospital managed CHCs was better than that of the other two models of CHCs [19]. The quality of health records established by the privately owned CHCs was the poorest among the three models [13]. The studies reported inconsistent results using hypertension control rate as a quality indicator, i.e. the proportion of hypertensive patients whose blood pressure levels were under the target (i.e., 140/90 mmHg). One study [13] showed that the hypertension control rate was similar among the three models of CHCs, while the other study [21] showed that the hypertension control rate was the highest among privately owned CHCs, followed by hospital managed CHCs and then government managed CHCs. These conflicting results might be due to the underlying patient population mix.

### Public satisfaction

The difference in public satisfaction regarding the primary care services provided by different models of CHCs was identified in six studies (Table 5). Four studies had shown that privately owned CHCs were more convenient than other models of CHCs [21, 26–28]. These studies also showed that more community residents were impressed by equipment in privately owned CHCs compared with the other two models of CHCs. Overall satisfaction was found to be the highest for government managed CHCs and the lowest for privately owned CHCs [21, 25, 26]. Community residents were more satisfied with the price of health services and drugs provided by government managed CHCs than that of the other models of CHCs, while they were more satisfied with the clinical services provided by hospital managed CHCs than other models [26, 27]. No conclusion could be drawn in terms of patient satisfaction regarding hospital environment and the attitude of health workers across the three models of CHCs. Patients were more satisfied with tertiary hospital managed CHCs compared with CHCs managed by smaller hospitals [29].

**Table 5:** Patient satisfaction ratings in proportions or scores

First author	Comparison	Convenience	Environment	Equipment	Attitude	Price	Skill	Safety	Overall
Zhao Kun^a^	G	94.6	90.6	73	93.3	76.7	78.4	78.4	84.1
	H	94.1	83.1	61	95.4	73.5	84.4	89.2	84.1
	P	95.8	88.7	74.6	90.2	67.6	73.3	70.5	80.6
Yao Hongxia^a^	G	95.59	91.18	72.06	94.12	79.41	80.88	82.35	-
	H	95.08	85.25	60.66	93.44	67.21	87.97	88.52	-
	P	96.92	92.31	78.46	92.31	72.31	76.92	73.85	-
Wang Hongzhi^a^	G + H	90.0	78.25	61.24	88.64	69.14	-	-	-
	P	94.91	87.39	73.64	93.13	66.48	-	-	-
Li Yongbin^a^	G + H	-	-	-	-	-	-	-	78.02
	P	-	-	-	-	-	-	-	72.71
Guo Haixiu^b^	G	83.4	79.8	71.4	90.8	78.2	83.6	-	85.4
	H	85.4	74.6	65.8	87.2	77.8	83.2	-	83.0
	P	86.4	79.0	73.0	87.0	78.0	83.6	-	84.4
Chen Jie^b^	H1	4.08	4.03	3.40	4.14	4.13	3.75	-	3.99
	H2	4.16	3.44	3.02	4.26	4.12	4.10	-	4.04
	H3	4.46	3.98	3.48	4.27	3.95	4.20	-	4.22

*Note*: *G* government managed CHCs, *H* hospital managed CHCs, *P* privately owned CHCs, *H1* primary hospital managed CHCs, *H2* secondary hospital managed CHCs, *H3* tertiary hospital managed CHCs

^a^in proportions

^b^in scores

## Discussion

This systematic review has retrieved and synthesized papers comparing the three models of CHCs, the main providers of primary care services in China. Even though a large number of studies of CHCs have emerged since 1997, studies that have given definite descriptions of the models of CHCs are relatively limited, and thus only 13 papers were included in this review. Privately owned CHCs were found to have the smallest workforce in healthcare, the lowest share of government funding against the total revenue, the highest share of out-of-pocket payments, and the lowest coverage rate of health insurance schemes. There was a general trend that the quality of primary care services provided by privately owned CHCs was the poorest among the three models of CHCs. Healthcare workers of privately owned CHCs received fewer training opportunities than the other two models of CHCs. Community residents tended to use more services from hospital managed CHCs, especially the tertiary hospital managed ones. Patients were mostly satisfied with privately owned CHCs as regards the convenience of their services and the medical equipments provided, while patients were more satisfied with the better health services provided by hospital managed CHCs.

### Limitations of the study

The present systematic review provides a summary of the differences among the three major models of CHCs in China, which may allow policy makers to develop strategies on health care resources allocation and care supervision. However, some limitations should be mentioned hereby. Firstly, this systematic review only included a small number of studies. The robustness of the current findings might be limited. For one thing, selection bias might have been introduced as only papers in English or Chinese were retrieved, though we believe this may cover most of studies on this topic. For another, inclusion bias might exist because most studies were excluded after reviewing titles and abstracts but not through full-text reviewing, which might have limited the generalizability of the study findings. A second limitation may relate to the methodological quality of included studies. Three studies employed purposive sampling method to select CHCs which might have introduced sampling bias although we considered this sampling strategy to be relevant to the objectives of the studies. Of the six studies with respect to patient satisfaction, few have reported the validity and reliability of the measures employed, although the measures were widely recognized to be effective in assessing patient satisfaction. Additionally, they reported little consideration of confounding variables, limiting internal validity of the studies. Finally, the functions of CHCs in different local contexts such as financing approach and human resources were subject to the local implementation of primary care, e.g., regulations. Overall, caution should be made when interpreting the findings.

### Possible explanations for the main findings

All CHCs faced a serious shortage of trained health workers in China [31]. The average education level of the health workers remained relatively low, possibly because most graduates with higher education level tended to join large-scale hospitals. Primary care organizations tended to have a healthcare workforce with poor academic backgrounds, insufficient practice guidelines, and unclear responsibilities compared to hospitals [32]. Private CHCs were less competitive compared with other CHCs as public organizations, which have better job security and welfare benefits in China. Therefore, privately owned CHCs were found to have the smallest number of health workers, who had received the least training opportunities, which is possibly because of financial constraints and the profit-driven objectives of the privately owned CHCs.

Primary care was inadequately funded by the government even though it had been set out as the foundation of the new comprehensive reform plan in China [33]. Privately owned CHCs were the least well funded due to their ownership status, in spite of the increased payments to CHCs for public health services provision in recent years [12]. Without sufficient government funding, CHCs did not have the motivation to provide public health services that do not generate economic returns [34]. Thus, CHCs, especially the private ones, may focus more on providing clinical care than public health services.

Health insurance schemes have become a larger payer of the primary care services provided in CHCs due to the rapid expansion of the Basic Medical Insurance Scheme for Urban Employees and Basic Medical Insurance Scheme for Urban Residents, which together covered a total of over 80 % of the urban population in China as of the end of 2008 [35]. It was more likely for private CHCs to be left out by public health insurance schemes due to their ownership status. The elders and migrants, the two vulnerable groups in the community, tended to visit CHCs due to their low price and convenience [36], but these groups of users were more likely to be uninsured [37]. Lower insurance coverage usually results in lower use of essential health services among the elders [38] and migrants [39], which may jeopardize the goal of equity in primary care services’ provision. This also explains the observation why out-of-pocket payments consisted of the largest proportion of total revenues in CHCs.

This review has shown that the quality of community health services was more likely to be poor at the privately owned CHCs. This finding is in line with the studies from other countries that quality of care is usually worse in privately owned health facilities when compared with that of publicly financed ones [40–43]. Human resources are widely recognized to be associated with the quality of community health services [44]. However, in our study, the difference in the capacity of the health workers (i.e., education level and professional title) among the three models of CHCs was not consistent. Poor financial investment is one of the most significant impediments to the delivery of primary care services and contributes to poor quality of care [45]. Thus, insufficient investment from the government for the provision of public health services may be an important reason for the poor quality of public health services offered by privately owned CHCs. Additionally, the profit seeking nature of privately owned CHCs is another possible reason of lower quality of care provided by these CHCs, as they are more likely to prescribe drugs to patients to make profits.

It has been observed that people tended to use community health services provided by hospital managed CHCs, especially the tertiary hospital managed ones. This reflects the better reputation of hospitals, especially big hospitals, regarding quality of health care [46]. Higher satisfaction with clinical services may also contribute to the higher utilization rate of primary care services offered by hospital managed CHCs. Moreover, the greater awareness of community health services provided by the hospital managed CHCs in the community may be another possible reason for the better utilization of hospital managed CHCs [29].

Privately owned CHCs had the lowest overall satisfaction ratings, which may possibly due to the perceived poor quality of health services, as with similar results found in other countries [47]. Private CHCs tend to compete with public ones on the convenience of services (i.e., opening hours and locations), the medical equipment and the appearance of offices [48].

### Policy implications

This systematic review provides comprehensive evidence to policy makers for the future development of health policies for better primary care system development in China. In response to health personnel shortage and their limited capacity, the State Council of the Central Government has enacted a plan with specific strategies in training both new graduates and the current health workforce. In addition, other policies should be targeted at better professional recognition and higher salaries through government funding. The capitation payments can encourage CHC doctors to provide care of good quality [49]. Special attention should be given to privately owned CHCs as international evidence has shown that private organizations are less likely to provide training for their employees [50-51]. Doctors in private CHCs are likely to have better capacity in primary care if the government purchases public health care services as it does from public ones. Health insurance schemes should cover private CHCs as well as public ones to ensure better financial access to primary care services [52], as vulnerable populations such as the elderly and migrants tend to use CHC services.

Research gaps have been identified regarding the performance of different primary care delivery models. Quality improvement is the central focus of health care [53]. Recent studies suggested a possible association between quality of care and organizational factors among primary care providers [54, 55]. CHCs of different models in China have different financing approaches, numbers in their health workforce, as well as premises; we speculate that these differences may translate into differences in the quality of primary care services supplied. However, a few studies compared the quality of care delivery processes using indicators such as the appropriateness of medication prescriptions, the completeness of health records and vaccination rates, all of which were from a health professional’s perspective. Few explored the quality of care delivery processes from a patient’s perspective for performance comparison among CHCs. No such studies developed a comprehensive framework for such a performance comparison among different models of CHCs, rendering it difficult to characterize how well each model performs across the dimensions of primary care (i.e., structure, process and outcome) and make it difficult to provide strategic recommendations for the long-term development of primary care.

## Conclusions

In summary, government and hospital managed CHCs in China were more competent and tended to provide primary care services with higher quality than privately owned CHCs. More studies are warranted to comprehensively address the performances of different models of CHCs.
